# Sintering Ag_33_ Nanoclusters on TiO_2_ Nanoparticles as an Efficient Catalyst for Nitroarene Reduction

**DOI:** 10.3390/ma17246120

**Published:** 2024-12-14

**Authors:** Weihua Zhang, Wenwen Yang, Jianglu Yuan, Huiping Zhao, Qingwen Han, Wanggang Fang, Defu Nie, Liqing He, Fan Tian

**Affiliations:** 1 Key Laboratory of Green Chemical Process of Ministry of Education, School of Chemistry and Environmental Engineering, Wuhan Institute of Technology, Wuhan 430205, China; 2 Hubei Three Gorges Laboratory, Yichang 443007, China; 3 Hefei General Machinery Research Institute Co., Ltd., Hefei 230031, China

**Keywords:** metallic nanoclusters, oxygen vacancies, selective hydrogenation, metal oxide

## Abstract

Polydispersed Ag species-modified TiO_2_ samples with abundant oxygen vacancies were successfully prepared through the calcination of atomically precise Ag_33_ nanocluster-loaded TiO_2_ at an optimal temperature under a nitrogen atmosphere. The ligands of the Ag_33_ nanoclusters are removed by extracting lattice oxygen from TiO_2_ during the calcination, leading to the formation of CO_2_, SO_2_, and H_2_O vapor. This process simultaneously induces Ag species sintering on the surface of TiO_2_. The resulting nanocomposites exhibited excellent catalytic activity for the reduction of nitroarenes with NaBH_4_ as the reductant. This is attributed to the produced Ag species on the oxygen-deficient TiO_2_, which act as active centers for the catalytic process.

## 1. Introduction

Selective hydrogenation of nitroarenes to anilines is an important reaction as the produced anilines are essential building blocks for the manufacture of fine chemicals, pigments, dyes, biochemicals, agrochemicals, and pharmaceuticals [1,2,3,4,5,6]. Commercial methods to achieve the reaction rely on the non-catalytic use of stoichiometric reducing agents such as sodium hydrosulfite, tin, iron, or zinc. However, these methods not only exhibit low conversion rates but also produce large amounts of solid waste that are difficult to handle. Although precious metal catalysts such as Pd, Au, and Ru have been widely explored in the reaction owing to their extraordinary catalytic actives, their high cost and low selectivity (when the nitroarene contains reducible groups such as C-Br, C=O, C=C, etc.) greatly hinder their large-scale applications. Therefore, exploring less precious metal-based catalysts with high efficiency and selectivity for the reaction is necessary.

Metallic nanoclusters with atomic precision have attracted increasing research interests in catalysis owing to their potential for elucidating precise correlations between structure and catalytic performance [7,8,9,10,11,12,13] and as building blocks for the design of highly efficient catalysts [14,15,16,17,18], which could deepen our understanding of the catalytic process at the molecular level and guide further exploration of highly efficient catalysts [19,20,21,22,23]. Employing nanoclusters as the catalyst for the reduction of nitroarenes, especially with NaBH_4_ as the reductant, has become a benchmark test to evaluate the catalytic activity of nanoclusters owing to the mild reaction conditions that can be conveniently carried out in a chemical synthesis laboratory [24,25,26,27,28,29]. In this scenario, nanoclusters with different protecting ligands, such as Au_11_ [19], Au_19_/Au_20_/Ag_22_ [30], Au_25_ [31,32], Au_28_ [32], Au_38_ [33], Ag_25_Cu_4_Cl_6_ [34], Ag_32_ [27], Pd_6_ [5], Pd_8_ [26], and others, have been explored as efficient catalysts for the reaction. The ligands on the nanoclusters were often considered bystanders in these systems, with the metallic cores being believed to serve as the primary active centers for the catalysis. However, our previous work utilizing a series of Ag_33_ nanoclusters capped by phenylethanethiol, benzyl mercaptan, and substituted benzyl mercaptan on TiO_2_ as heterogeneous catalysts revealed that the ligands of the nanoclusters could regulate their catalytic activity by imposing π–π interaction on the reactant [25]. After removing the ligands through pyrolysis, we observed an unexpected increase in catalytic activity. It suggests the formation of more active species during the pyrolysis process, but this aspect was not addressed in the work as it was beyond the research scope. Herein, we systematically detail the structural evolution of Ag_33_ nanoclusters on a TiO_2_ support during calcination. Experimental results revealed that the Ag_33_ nanoclusters sintered on the TiO_2_ support, with the nanocluster ligands extracting lattice oxygen from the support, producing CO_2_, SO_2_, and H_2_O vapor. The process resulted in oxygen-deficient TiO_2_ samples with in situ deposition of polydispersed Ag clusters, which significantly enhanced the catalytic activity.

## 2. Experimental

### 2.1. Materials

Silver nitrate (AgNO_3_, AR) and sodium borohydride (NaBH_4_, AR) were purchased from YongHua Chemical (Suzhou, China). Triphenylphosphine (PPh_3_, 99%), methanol (MeOH, AR), dichloromethane (CH_2_Cl_2_, AR), trichloromethane (CHCl_3_, AR), and hexane (C_6_H_14_, AR) were obtained from Sinopharm (Shanghai, China). Nano-TiO_2_ (P25, 99.8%) was purchased from EVONIK Degussa (Essen, Germany). 4-Chlorobenzylmercaptan (ClPhCH_2_SH, 98%) and tetrakis (triphenylphosphine) palladium (Pd(PPh_3_)_4_, 99%) were purchased from Energy Chemical (Shanghai, China). Ultrapure water was used in all experiments. All reagents were used directly without further purification.

### 2.2. Methods

***Synthesis of Ag_33_(p-BMTC)_24_(PPh_3_)_4_*** *(**p-BMTC=4-chlorobenzylmercaptan**) **nanoclusters:*** The synthesis of Ag_33_(p-BMTC)_24_(PPh_3_)_4_ (abbreviated as Ag_33_) nanoclusters was conducted according to our previously reported method [10,25]. In the typical synthesis, 1.2 g of AgNO_3_, 0.25 g of Pd(PPh_3_)_4_, and 5 g of PPh_3_ were dispersed in a mixture of 100 mL of methanol and 400 mL of dichloromethane under vigorous stirring. Once the solution became clear, 1.5 mL of 4-chlorobenzyl mercaptan was added. The solution was stirred for an additional 60 min to allow the metal atoms to fully coordinate with the ligands. Then, 10 mL of an aqueous NaBH_4_ solution (60 mg/mL) was added in four portions, each measuring 2.5 mL. A purplish red solution was obtained after maintaining the reaction at 10 °C for 24 h. Purified Ag_33_ nanoclusters were gained by rotary evaporation and centrifugation. Dark Ag_33_ single crystals were grown by layering hexane into the saturated CHCl_3_ solution of the purified products at 4 °C for one week.

***Loading Ag_33_(p-BMTC)_24_(PPh_3_)_4_ nanoclusters on TiO_2_ support:*** Loading the Ag_33_ nanoclusters on TiO_2_ was simply conducted via an evaporation method. In the preparation, 10 mg of Ag_33_ crystals were dissolved in 10 mL dichloromethane to form a clean solution. After adding 100 mg of TiO_2_ powder, the solution was sealed into a rotary evaporator with a bath temperature of 30 °C to allow the dichloromethane to evaporate completely. The obtained solid was then collected and ground as the target Ag_33_-loaded TiO_2_ (abbreviated as Ag_33_/TiO_2_) products.

***Transforming Ag_33_-loaded TiO_2_ to the Ag_33_-sintered TiO_2_ samples:*** First, 100 mg of the Ag_33_/TiO_2_ powder was placed in a tubular furnace with a diameter of 50 mm and heated to 400 °C for 2 h at a heating rate of 5 °C/min under nitrogen gas (100 mL/min). After calcination, the samples were cooled to room temperature to obtain Ag_33_-sintered samples. Other samples were obtained under identical conditions with different calcination temperatures. All the samples were labeled by their calcination temperature.

***Catalytic reduction of 4-nitrophenol:*** First, 1 mg of catalyst was well dispersed in 5 mL of 4-nitrophenol (500 mg/L) aqueous solution under ultrasonication. Then, 15 mL of an aqueous solution containing 75 mg of NaBH_4_ was added to the mixture under ultrasonication. At each given time interval, 0.5 mL of the suspension was collected and filtered with a 0.45 μm MCE filter. The concentration of 4-nitrophenol in the solution during the catalysis was monitored by its characteristic absorption peaks at 400 nm with a UV-vis spectrophotometer. All the measurements were carried out at room temperature.

***Characterization:*** The UV-vis absorption spectra of the samples were recorded using a V-1800 (AOE Instruments, Shanghai, China) spectrophotometer. Atomic absorption spectroscopy (AAS) was performed using an SP-35200AA (Shanghai Spectrum, Shanghai, China) atomic absorption spectrometer. X-ray diffraction (PXRD) was recorded on a Bruker axs D8 Discover (Bruker, Karlsruhe, Germany) diffractometer (Cu K*α* = 1.5406 Å) at a scanning rate of 10°/min. TEM and SEM images of the samples were recorded in Talos F200X and FEI Sirion electron microscopes(Thermo Scientific, Waltham, MA, USA), respectively. High-angle annular dark-field (HAADF) images were acquired on an FEI Titan 80–300 scanning transmission electron microscope (Thermo Scientific, USA) equipped with a probe spherical aberration corrector. X-ray photoelectron spectroscopy (XPS) was performed on an ESCALAB XI+ (Thermo Scientific, USA) photoelectron spectrometer with Al K*α* as the X-ray source. All spectra were calibrated using the C 1s peak at 284.8 eV. The Brunauer–Emmett–Teller (BET) specific surface area of the powders was analyzed by nitrogen adsorption using a Micromeritics ASAP 2020 nitrogen adsorption apparatus (Micromeritics, Norcross, GA, USA).

***Computational details:*** DFT calculations were performed using the CP2K package (version 2024.2) [35]. The wave functions were expanded in molecular optimized double-ζ Gaussian basis sets (DZVP-MOLOPT-GTH) with an auxiliary plane wave basis set with a cutoff energy of 300 Ry and a rel_cutoff energy of 45 Ry. Core electrons were modeled by scalar relativistic norm-conserving pseudo potentials with valence electrons of 11, 6, 4, 5, 6, 12, and 1 for Ag, S, C, P, O, Ti, and H, respectively. The DFT-D3 van der Waals correction by Grimme was applied to describe weak interactions in the system. The structure of the Ag_33_ nanoclusters was constructed based on our previously reported molecule structure derived from single-crystal X-ray diffraction [25], with the organic groups connected with P or S atoms simplified using a methyl group to reduce the computational cost. The as-obtained structure was then placed in the vacuum layer of a TiO_2_ slab model with the (001) facet exposed, which was constructed with the VESTA (version 3.5.7) [36] and Avogradro 1.9.4 [37] software packages. The parameters of the slab model were a = b = 26.495 Å, c = 60 Å, and α = β = γ = 90 °C. Brillouin zone integration was performed with a reciprocal space mesh consisting of only the gamma point. All the ab initio molecular dynamics (AIMD) were implemented at 1 fs per step under 300 K with the NVT ensemble. A heating bath with the Nosé−Hoover method was employed to control the temperature of the system. The energy convergence criterion of the DFT calculation in the AIMD was 3 × 10^−5^ hartree. Geometry optimizations of the systems were performed with a DFT energy convergence criterion of 3 × 10^−7^ hartree until the forces acting on each atom were below 0.00025 eV/Å by using the wavelet Poisson solver with the Broyden–Fletcher–Goldfarb–Shanno (BFGS) algorithm. All the calculations were performed under periodic boundary conditions, applied along the XY plane. The calculated results were visualized with the Mercury 2024 or VMD 1.9.4 package [38]. 

## 3. Results and Discussion

**Figure 1:**
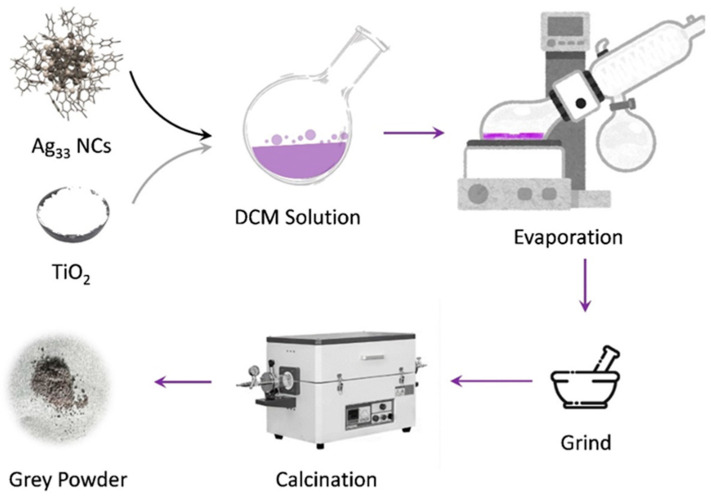
Illustration of the preparation of Ag_33_-sintered TiO_2_ samples.

Figure 1 illustrates the schematic representation of the preparation process for TiO_2_ samples sintered with Ag_33_ nanoclusters. The Ag_33_ nanocluster co-protected by triphenylphosphine and 4-chlorobenzylmercaptan was selected as the precursor owing to its relatively good stability. Unlike the Ag_33_ nanocluster synthesized with 2-phenylethanethiol, which requires additional triphenylphosphine for stabilization, this nanocluster can be stably dissolved in pure dichloromethane (DCM), resulting in a violet solution with two absorption bands centered at 533 nm and 475 nm (Figure S1), which is in agreement with previous reports [25]. After adding a typical amount of commercial TiO_2_ (P25, Degussa), the obtained solution was then sealed into a rotary evaporator to allow the solvent to evaporate completely. This process resulted in mauve solid products stuck on the flask. The obtained products were then collected and ground to produce Ag_33_-loaded TiO_2_ (abbreviated as Ag_33_/TiO_2_) samples. In these samples, the Ag_33_ nanoclusters interacted weakly with the TiO_2_ surface due to the presence of the ligands. Removing the ligands could be accomplished by calcining the samples in a tube furnace under the flow of nitrogen gas, which eventually generated a grey powder.

**Figure 2:**
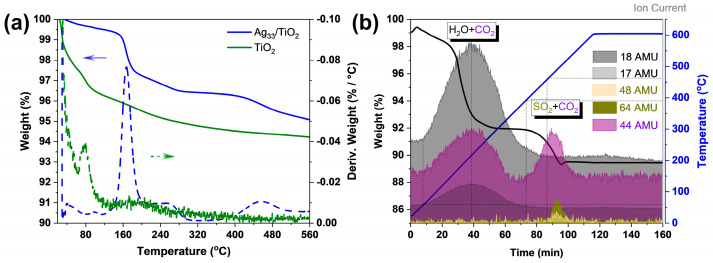
Comparison of the TG analysis of TiO_2_ and Ag_33_/TiO_2_ samples (**a**) and the TG-MS spectrum of the Ag_33_/TiO_2_ samples (**b**).

To understand the ligand removal of the Ag_33_ nanoclusters on TiO_2_ samples during the calcination, the freshly prepared Ag_33_-loaded TiO_2_ samples were characterized with thermogravimetric analysis (TGA) and thermogravimetric mass spectroscopy (TG-MS) under a N_2_ atmosphere. The TGA results show that there are two major thermogravimetric signals which begin at 150 °C and 400 °C (Figure 2a). The removal of adsorbed H_2_O (~5 wt%) on the Ag_33_-loaded TiO_2_ during the analysis occurs below 100 °C, as evidenced by comparing with the TGA of TiO_2_, where the primary mass loss is observed around 80 °C. The TG-MS results show that the weight loss of the Ag_33_-loaded TiO_2_ occurs in two distinct periods. The first period, from 60 to 350 °C, is associated with the volatilization of H_2_O and CO_2_, while the second period, from 380 to 550 °C, is characterized by the volatilization of CO_2_ and SO_2_ (Figure 2b). No distinguishable signals associated with phosphine were observed, which might be ascribed to its low content (the theoretical mole ratio of P/S is 1/6). As the analysis was performed under a N_2_ atmosphere, the volatilization of H_2_O, CO_2_, and SO_2_ indicates that the lattice oxygen atoms of TiO_2_ were partially stripping out, resulting in the oxidation of the ligands. The first weight loss period of approximately 6 wt% reached its maximum at around 200 °C, mainly due to the removal of alkane chains in the ligands of Ag_33_ nanoclusters. In contrast, the second period only resulted in a 2.5 wt% weight loss, occurring at an optimal temperature of around 500 °C. This loss, we believe, is associated with the remaining C-S species that were strongly bonded to the surface of TiO_2_ or to the Ag species that were produced during the previous period. It indicates that the removal of ligands from the Ag_33_ nanoclusters on the surface of TiO_2_ follows two steps rather than a single indistinctive step. Based on these results, it can be concluded that calcining the Ag_33_-loaded TiO_2_ samples at different temperatures could result in oxygen-deficient TiO_2_ samples modified with different Ag species. These observations motivated us to adjust the calcination temperature to obtain different Ag-species-sintered TiO_2_ samples, allowing us to identify which specific Ag species is most advantageous for catalysis.

**Figure 3:**
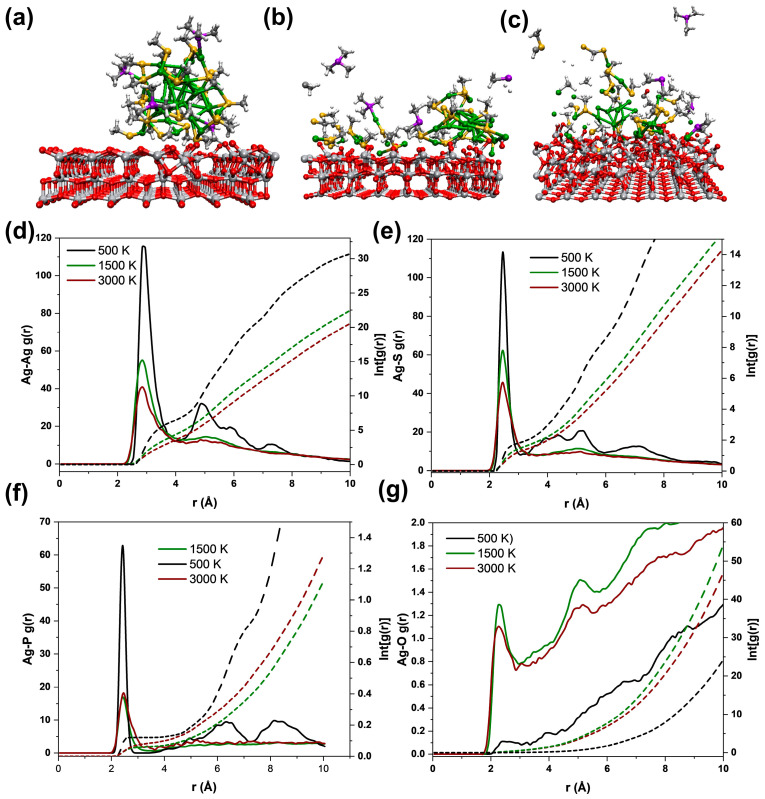
Snapshots from the AIMD of Ag_33_ on the (001) facet of TiO_2_ at 500 K (**a**), 1500 K (**b**), and 3000 K (**c**) after the systems reached equilibrium, along with the corresponding pair distribution function (the solid line) and its integral (the dash line) for Ag-Ag (**d**), Ag-S (**e**), Ag-P (**f**), and Ag-O (**g**) of these systems.

In order to understand the ligand removal of the Ag_33_ nanoclusters on the TiO_2_ and illustrate the surface structure evolution of the TiO_2_ samples, we performed ab initio molecular dynamics (AIMD) simulations at different temperatures for the interacting systems. To reduce computational costs, the alkyl groups in the ligands of the Ag_33_ nanoclusters were simplified with methyl groups. The initial structure of the simulation was constructed by locating the simplified Ag_33_ nanocluster in a slab model of TiO_2_ with the (001) facet exposed (Figure S2). The results show that all the systems reached equilibrium after a certain simulation time, with temperatures eventually fluctuating around the set value (Figure S3a–c). The continuous decrease in energy for these systems demonstrates that the structural evolution of the clusters on the surface of TiO_2_ is energetically favorable. The snapshot of the systems after equilibrium at 500 K (Figure 3a) shows that the main structure of the Ag_33_ was persistent during the simulation, which can be a benchmark for analyzing the structural evolution of the clusters on TiO_2_ at higher temperatures. The temperature was raised to unrealistic values of 1500 K and 3000 K to accelerate the simulations, allowing us to capture key processes related to ligand removal and the sintering of Ag species, which occur in experiments. The corresponding snapshots (Figure 3b,c) show that ligand removal from Ag_33_ leads to the degradation of the nanocluster (for detailed information, refer to the Supporting Videos). Typically, phosphine ligands detach from the nanocluster as a whole, while thiol ligands evolve with Ag atoms by removing their alkyl groups and forming Ag-S-Ag species on the surface of TiO_2_ (Figure 3b). With higher temperatures, the S atoms can be removed from the Ag species by forming thioether (Figure 3c). These species were absent in the TG-MS, which might be due to their low concentration. The observed CO in the snapshot shows that the removal of the ligands can be theoretically realized by extracting oxygen on the TiO_2_ surface. The pair distribution function (PDF) derived from the AIMD can provide the statistical information of the evolutions. We then compared the PDF between different atoms for these systems. The distinguishable multi-peaks below 10 Å for Ag-Ag (quadruple, Figure 3d, black line), Ag-S (quadruple, Figure 3e, black line), and Ag-P (triple, Figure 3f, black line) in the system at 500 K correlate to the topological structure of Ag_33_ nanoclusters. The integrals for the first peak are 6 and 2 for Ag-Ag (Figure 3d, black dashed line) and Ag-S (Figure 3e, black dashed line), indicating close contact between them during the simulation. The integral of Ag-P for the first peak, however, is about 0.2, which is far below 1 (a P atom is coordinated with one Ag atom), demonstrating that the phosphine ligand of the Ag_33_ nanocluster is susceptible. Raising the temperature to 1500 K and 3000 K not only reduces the intensity of the first peaks but also makes the rest of the distribution flat (Figure 3d–f, green and red lines), which correlates to the destruction of the structure of the Ag_33_ nanocluster. Particularly, the Ag-O PDF distribution for the system at 500 K shows no distinguishable peaks and is almost negligible compared to the system at 1500 K and 3000 K. The presence of a peak at 2.5 Å for the systems at 1500 K and 3000 K indicates the formation of an Ag-O bond on the TiO_2_ surface. These results suggest that the ligand removal of the Ag_33_ nanocluster on TiO_2_ is initially accompanied by the structural degradation of the cluster through the formation of Ag-O bonds. This process bridges the spatial gap between the surface of TiO_2_ and the cluster ligands, facilitating the interaction of lattice oxygen with organic species to generate CO_2_, SO_2_, and H_2_O.

**Figure 4:**
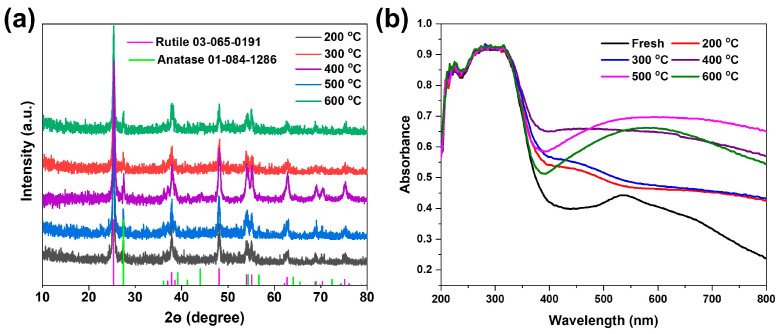
Powder X-ray diffraction (**a**) and the UV-vis diffuse reflection spectra (**b**) of the Ag_33_/TiO_2_ samples after calcination under different temperatures.

Based on the above results, we then calcined the Ag_33_-loaded TiO_2_ samples starting at 200 °C and at intervals of 100 °C under a nitrogen atmosphere to obtain different samples. Powder X-ray diffraction (PXRD) results show that all the obtained samples are composed of anatase and rutile, with no impurity peaks observed, demonstrating that the Ag species were not crystalized as large particles on the TiO_2_ samples (Figure 4a). As the Ag_33_ nanoclusters exhibited distinct optical absorption in the visible range, we then collected the UV-visible diffuse reflection spectra of the as-obtained samples. Pure TiO_2_ is a UV-responsive semiconductor with an absorption edge below 420 nm, meaning it cannot absorb visible light. When loaded with Ag_33_ nanoclusters, broad absorption in the visible range with a characteristic peak at ~540 nm is clearly observed (Figure 4b, black line). The absorption primarily corresponds to the Ag_33_ nanoclusters in the solution (Figure S1), suggesting that the structure of the nanoclusters is preserved on the TiO_2_ surface. However, calcining the samples at 200 °C or 300 °C caused a change in the peak shape, with the characteristic peak degrading and a new weak absorption feature appearing around 430 nm (Figure 4b, red and blue lines). This absorption is similar to the plasmon resonance effect observed in metallic Ag nanoparticles, suggesting that the removal of ligands at low temperatures may cause the nanoclusters to fuse into larger particles. Raising the temperature to 400 °C led to a flattening of the visible absorption, accompanied by an increase in absorption intensity (Figure 4b, violet line). We attribute the changes to the combined effects of Ag clusters sintering on the TiO_2_ surface and oxygen vacancy formation in its subsurface. Further increasing the temperature of the calcination to 500 °C and 600 °C resulted in an absorption decrease near the band edge of TiO_2_, while the following visible absorption was enhanced. These results indicate that the removal of ligands from Ag_33_ nanoclusters on the surface of TiO_2_ at different temperatures leads to the formation of Ag species-sintered TiO_2_ samples with different surface characteristics.

**Figure 5:**
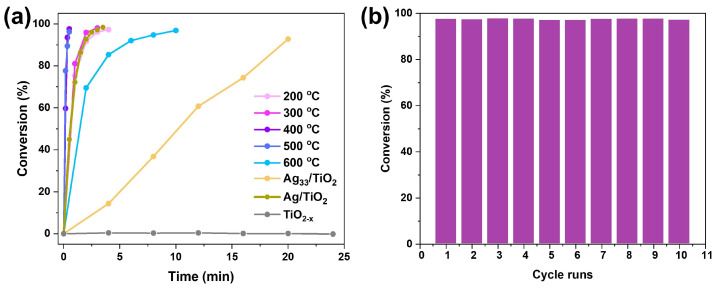
The catalytic activity of Ag_33_/TiO_2_ after treatment at different temperatures (**a**) for 4-NP reduction and the reusability evaluation of the Ag_33_/TiO_2_ samples after 400 °C treatment (**b**).

The catalytic activity of the as-obtained samples for nitroarene reduction with NaBH_4_ as the reductant was evaluated by using 4-nitrophenol (4-NP) as the target reactant. The Ag_33_ nanocluster-loaded TiO_2_ samples exhibited intrinsic activity in the conversion of 4-nitrophenol (4-NP) to 4-aminophenol (4-AP) within 20 min (Figure 5a), which is evidenced by the changes in their characteristic peaks at 400 nm and 300 nm, respectively (Figure S4a). Calcining the samples at varying temperatures led to an overall enhancement in catalytic activity. Control experiments showed that pure TiO_2_ with oxygen vacancies (TiO_2−x_) is completely inert for catalysis, while the catalytic activity of the TiO_2_ with Ag (Ag/TiO_2_) prepared by the impregnation method is almost comparable to the as-prepared samples at 200 °C and 300 °C, which indicates that the Ag atoms exposed on TiO_2_ are the active sites for the catalysis. Notably, the catalytic activity increased with temperature, reaching its maximum at 400 °C, where complete conversion of 4-NP was achieved in only 30 s (Figure 5a and Figure S4b–f). Further increasing the temperature to 600 °C resulted in a decline in catalytic activity, indicating that excessively high calcination temperatures are not beneficial for catalysis. This may be due to the elevated temperature promoting the sintering of Ag species into the subsurface of TiO_2_, thereby reducing their availability as active centers for the reaction. Based on the results, we conclude that the optimal calcination temperature for Ag_33_-loaded TiO_2_ samples to generate a highly efficient catalyst for nitroarene reduction is 400 °C. The cyclic activity evaluation revealed that the as-obtained samples maintained their catalytic efficiency even after being used nine times, demonstrating impressive reusability (Figure 5b).

**Figure 6:**
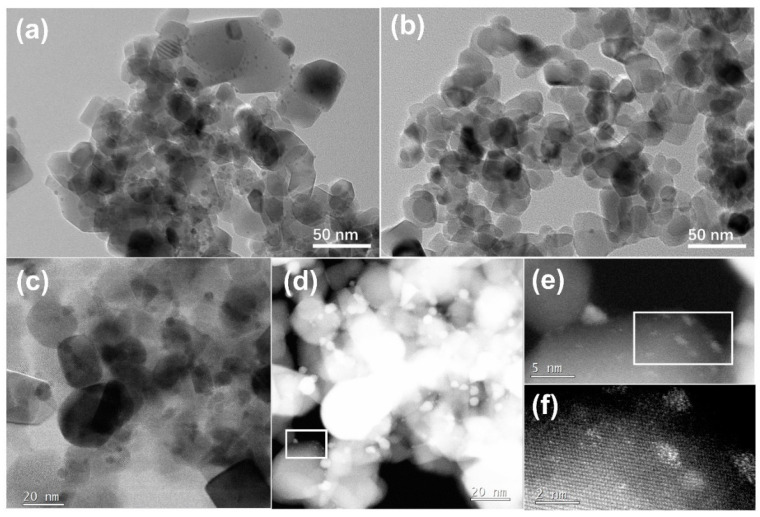
TEM images of the Ag_33_/TiO_2_ before calcination (**a**) and after calcination at 400 °C (**b**,**c**), along with the corresponding HAADF images for the samples after calcination (**d**–**f**).

To better understand how calcination yields highly efficient catalysts for the nitroarene reduction, we characterized the Ag_33_-loaded TiO_2_ samples before and after calcination at 400 °C with a transmission electron microscope (TEM). The obtained TEM image of the Ag_33_-loaded TiO_2_ samples reveals a particle morphology with diameters ranging from 30 to 60 nm, consistent with prior reports on commercial TiO_2_ supports [25]. Additionally, numerous nanodots, each less than 5 nm in diameter, are evenly distributed on the surface of these TiO_2_ particles (Figure 6a), corresponding to the Ag_33_ nanoclusters. These nanodots became invisible after the samples were calcinated (Figure 6b,c), demonstrating that the structure of the Ag_33_ was destroyed. The corresponding HAADF images of the samples (Figure 6d–f) indicate that calcination caused the Ag_33_ nanoclusters to decompose into polydispersed Ag species, including nanoparticles, clusters, and even single Ag atoms anchored on the surface of TiO_2_. It shows that calcination induces the structural evolution of the nanoclusters by removing their organic ligands and reconstructing the metallic core, leading to the sintering of polydispersed Ag species on the surface of TiO_2_ as the active species for the catalytic nitroarene reduction.

**Figure 7:**
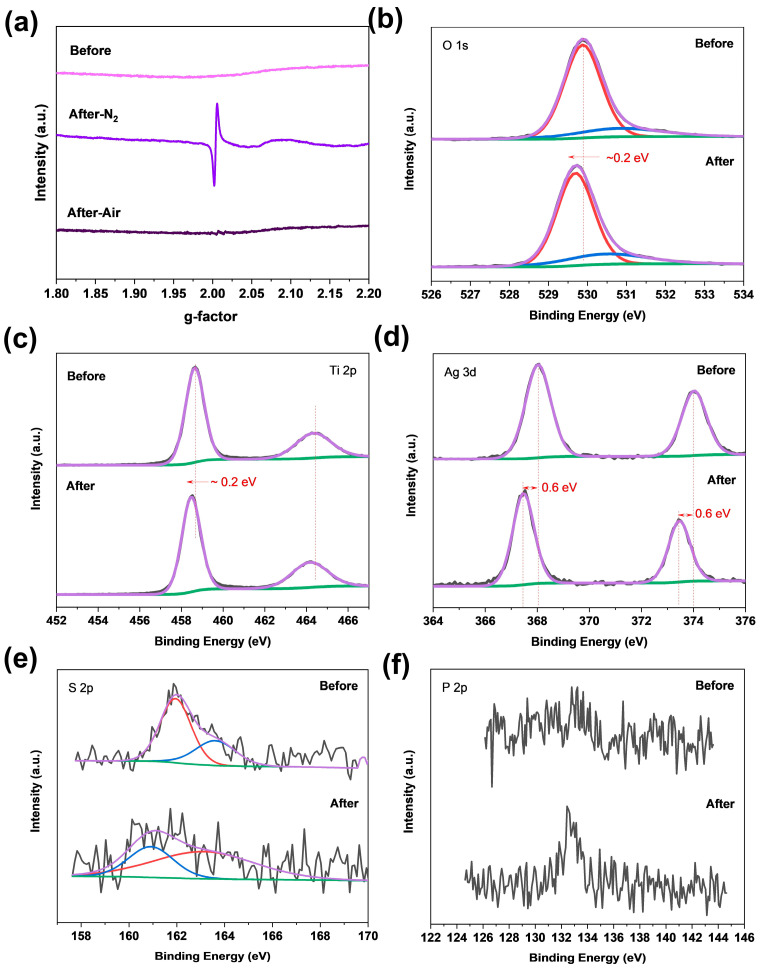
EPR (**a**) and high-resolution XPS spectra of O 1s (**b**), Ti 2p (**c**), Ag 3d (**d**), S 2p (**e**), and P 2p (**f**) of the Ag_33_-loaded TiO_2_ before and after calcination.

In order to prove the process, we further conducted EPR and XPS analyses of the Ag_33_-loaded TiO_2_ samples before and after calcination. The EPR spectra of pure TiO_2_ calcined under air atmosphere are almost identical to those of the Ag_33_-loaded TiO_2_ samples before calcination, with no distinct signal observed (Figure 7a), indicating that calcining the Ag_33_-loaded TiO_2_ samples under air atmosphere could not induce the removal of the lattice oxygen of the samples. In contrast, an obvious unpaired electron signal at g = 2.003 is observed for the Ag_33_-loaded TiO_2_ samples after calcination under a N_2_ atmosphere. This characteristic EPR signal corresponds to the oxygen vacancies of TiO_2_, showing that calcination in the presence of Ag_33_ nanoclusters results in the removal of oxygen from the lattice of the TiO_2_, which is in accordance with the formation of H_2_O, CO_2_, and SO_2_ observed in the TG-MS results. It indicates that the ligand removal from the Ag_33_ nanoclusters is accomplished by extracting the lattice oxygen of TiO_2_, which results in the decomposition of the ligands to form the gas spilling out. Survey XPS spectra show that both the samples are predominantly composed of Ti, O, C, and Ag elements, while S and P elements are almost invisible due to their low concentration on the surface of the TiO_2_ (Figure S5a). The Ag/Ti atomic ratio from the spectra of calcined samples is nearly half that of the freshly prepared ones (Table S1), indicating that ligand removal during calcination is accompanied by a loss of Ag atoms on the surface of TiO_2_. Typically, the C 1s signals of the samples, both before and after calcination, can be deconvoluted into two components. We attributed the low-binding-energy component to adventitious carbon contamination in the spectrometer. This peak (284.6 eV) was used to calibrate the XPS spectra, ensuring reliable binding energy values for the sample elements and enabling precise evaluation of the chemical shifts induced by calcination. The calcination induced a red shift of approximately 0.2 eV for the binding energies of O and Ti. Furthermore, the Ag 3d signal for the samples after calcination shows a red shift as large as ~0.6 eV compared to the freshly prepared Ag_33_-loaded TiO_2_ samples. It indicates that the produced Ag species are different to the metallic core of Ag_33_ nanoclusters. Upon removal of the ligand, the large red shift in the Ag 3d spectra is in accordance with the transformation of S-Ag-S to S-Ag-O or O-Ag-O [39,40]. It suggests that the metallic core of the Ag_33_ nanoclusters undergoes interaction with the exposed oxygen of TiO_2_, resulting in the formation of polydispersed Ag_x_-O species anchored on the surface, consistent with the AIMD simulations. The decreased intensity of S 2p spectra (Figure 7e) for the samples after calcination further confirms the removal of thiol ligands. Due to the low content of phosphine ligands in the samples, the high-resolution P 2p spectra failed to provide discernible information because of the low signal-to-noise ratio (Figure 7f). Based on these results and our previous related work [28,29], we tentatively conclude that the high efficiency for the catalytic nitroarene reduction of the Ag_33_-loaded TiO_2_ samples after calcination is ascribed to the presence of Ag species derived from the Ag_33_ nanoclusters by removing its ligands. Our previous work showed that Ag_4_M_2_ (M = Pd, Ni) nanoclusters on TiO_2_ or SiC can be in situ converted to metallic Ag particles, which accelerates the catalytic hydrogenation of nitroarenes [28,29]. To illustrate whether the catalytic process is driven by the Ag species or their potential reduced products, we further compared the XPS spectra of the Ag_33_-sintered samples before and after the reaction. The results show that the Ag 3d spectra are nearly identical before and after catalysis (Figure S6a,b), indicating that the ultrasmall Ag species on TiO_2_ are not reduced to metallic Ag particles during the catalysis. The slight blue shifts of ~0.1 eV for Ti 2p (Figure S6c) and O 1s (Figure S6d) also demonstrate the stability of the samples during catalysis. These results, which correlate with the insignificant activity of the oxygen-deficient TiO_2_, indicate that the Ag species are efficient active sites for the reduction of nitroarenes.

## 4. Conclusions

In summary, a novel Ag species-modified TiO_2_ sample with abundant oxygen vacancies was successfully obtained by calcining ligand-protected Ag_33_ nanocluster-loaded TiO_2_ samples at an appropriate temperature under nitrogen atmosphere. The ligands of Ag_33_ nanoclusters are eliminated by extracting lattice oxygen from TiO_2_ during calcination, leading to the production of CO_2_, SO_2_, and H_2_O vapor. The process involves Ag species interacting with the surface of the oxygen-deficient TiO_2_ nanoparticles, which can be reduced to metallic Ag species in situ, serving as active centers to drive the catalytic reduction of nitroarenes. This work presents a facile calcination method to fabricate metal species-loaded metal oxide nanocomposites with abundant oxygen vacancies, using ligand-protected metallic nanoclusters as precursors, which may guide the further development of highly efficient catalysts.

## Data Availability

The original contributions presented in the study are included in the article/Supplementary Materials, further inquiries can be directed to the corresponding authors.

## Supplementary Materials

The following supporting information can be downloaded at: https://www.mdpi.com/article/10.3390/ma17246120/s1, Figure S1: UV-visible adsorption; Figure S2: Initial AIMD structure; Figure S3: Temperature and energy changes during AIMD; Figure S4: Time-dependent UV-visible spectra for the catalysis; Figure S5: Survey and C 1s XPS; Figure S6: XPS before and after catalysis; Table S1: Quantification analysis derived from survey XPS; Videos S1~S3: AIMD trajectory at 500, 1500 and 3000 °C.
