# Supplementary material for: Sintering Ag33 Nanoclusters on TiO2 Nanoparticles as an Efficient Catalyst for Nitroarene Reduction
Source: Materials (Basel). 2024 Dec 14;17(24):6120. doi: 10.3390/ma17246120 (PMC11677127; doi:10.3390/ma17246120)
Supplement: Supplementary file 1 [file materials-17-06120-s001.zip › materials-3309192-supplementary.pdf]

Supplementary Materials

for

Sintering Ag_{33} Nanoclusters on TiO_2 Nanoparticles as an Efficient Catalyst for Nitroarene Reduction

Weihua Zhang [1,†], Wenwen Yang [1,†], Jianglu Yuan [1], Huiping Zhao [1,*], Qingwen Han [2], Wanggang Fang [3], Defu Nie [3], Liqing He [3] and Fan Tian *

[1] Key Laboratory of Green Chemical Process of Ministry of Education, School of Chemistry and Environmental Engineering, Wuhan Institute of Technology, Wuhan 430205, China;
[2] Hubei Three Gorges Laboratory, Yichang 443007, China
[3] Hefei General Machinery Research Institute Co., Ltd., Hefei 230031, China
***** Correspondence: hpzhao_yy@hotmail.com (H.Z.); tf@wit.edu.cn (F.T.)
[†] These authors contributed equally to this work.

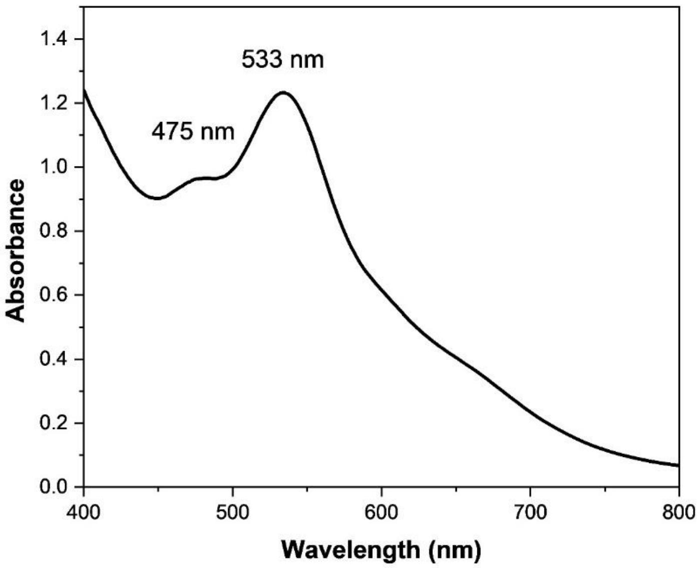

Figure S1 UV-visible absorption spectra of Ag_{33} synthesized from 4-chlorobenzylmercaptan and triphenylphosphine dissolved in dichloromethane.

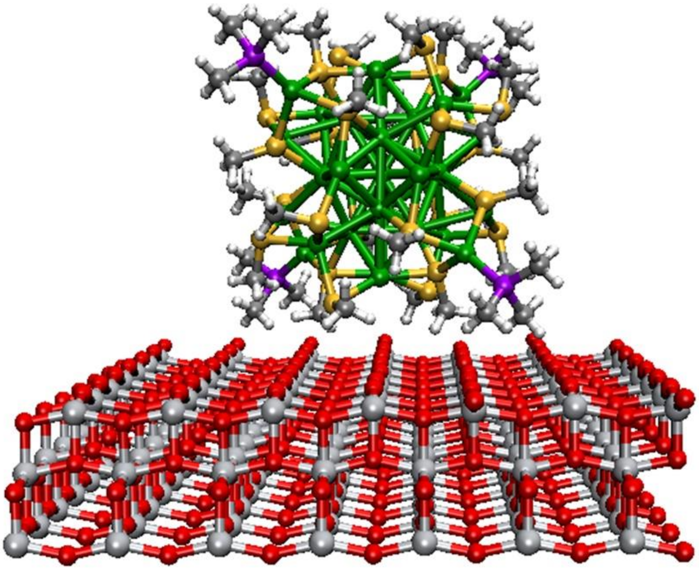

Figure S2 The initial structure of AIMD for the Ag_{33} loaded TiO_2 sample.

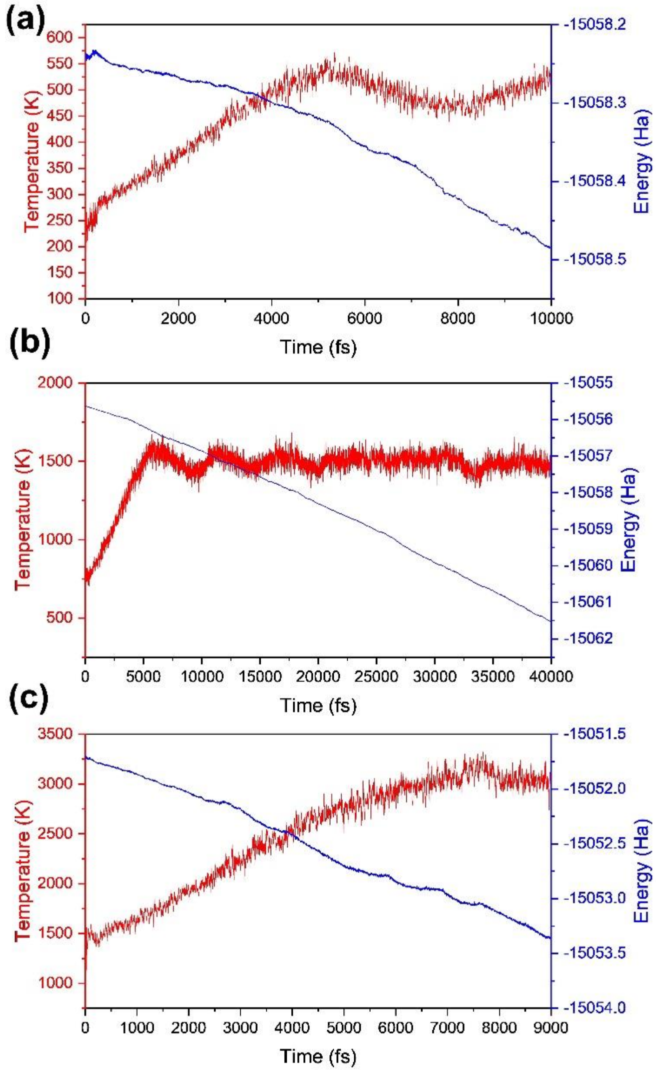

Figure S3 Temperature and energy vs. time plot of the AIMD for the Ag_{33} loaded TiO_2 sample at 500K, 1500K and 3000K.

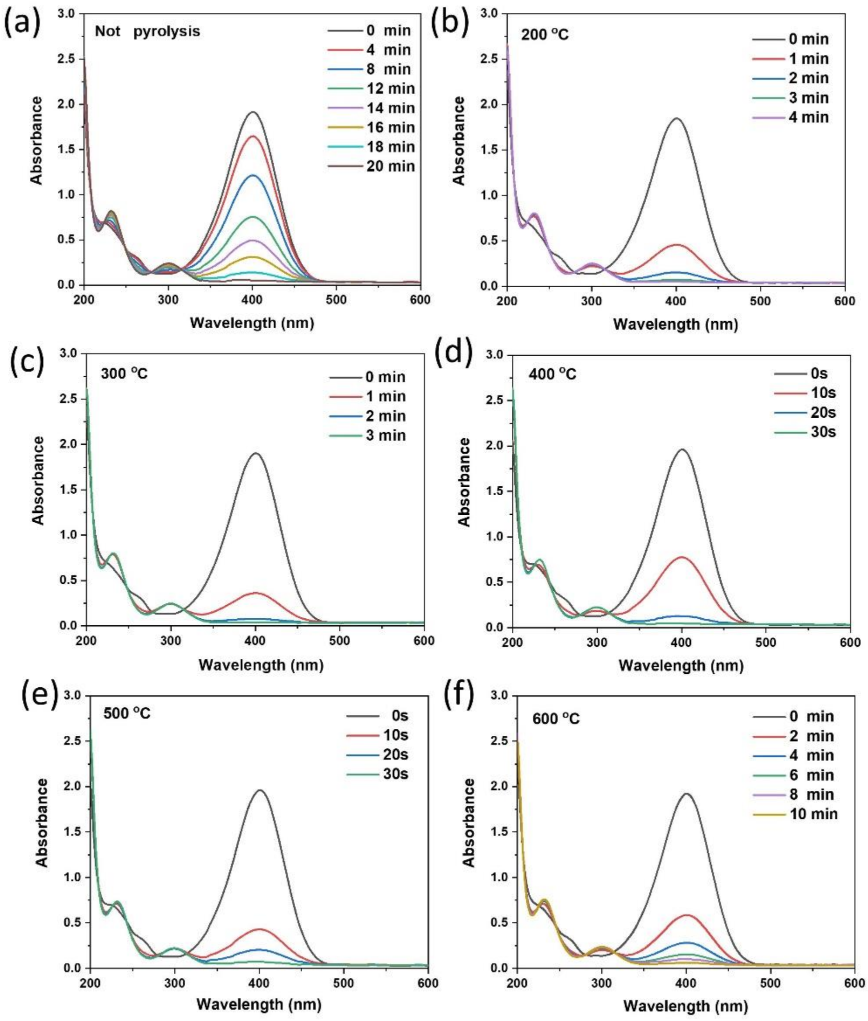

Figure S4 Time-dependent UV-vis spectra for the 4-nitrophenol reduction catalyzed by Ag_{33} loaded TiO_2 samples (a) and the samples after 200 ºC (b), 300 ºC (c), 400 ºC (d), 500 ºC (e) and 600 ºC (f).

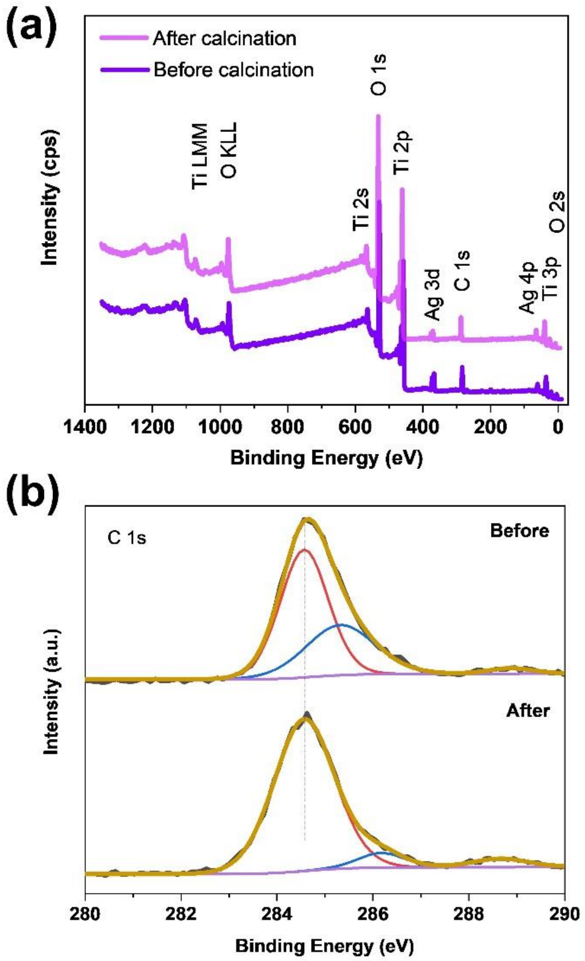

Figure S5 Survey XPS (a) and high-resolution C 1s spectra (b) of Ag_{33} loaded TiO_2 before and after calcination at 400 °C under N_2 atmosphere.

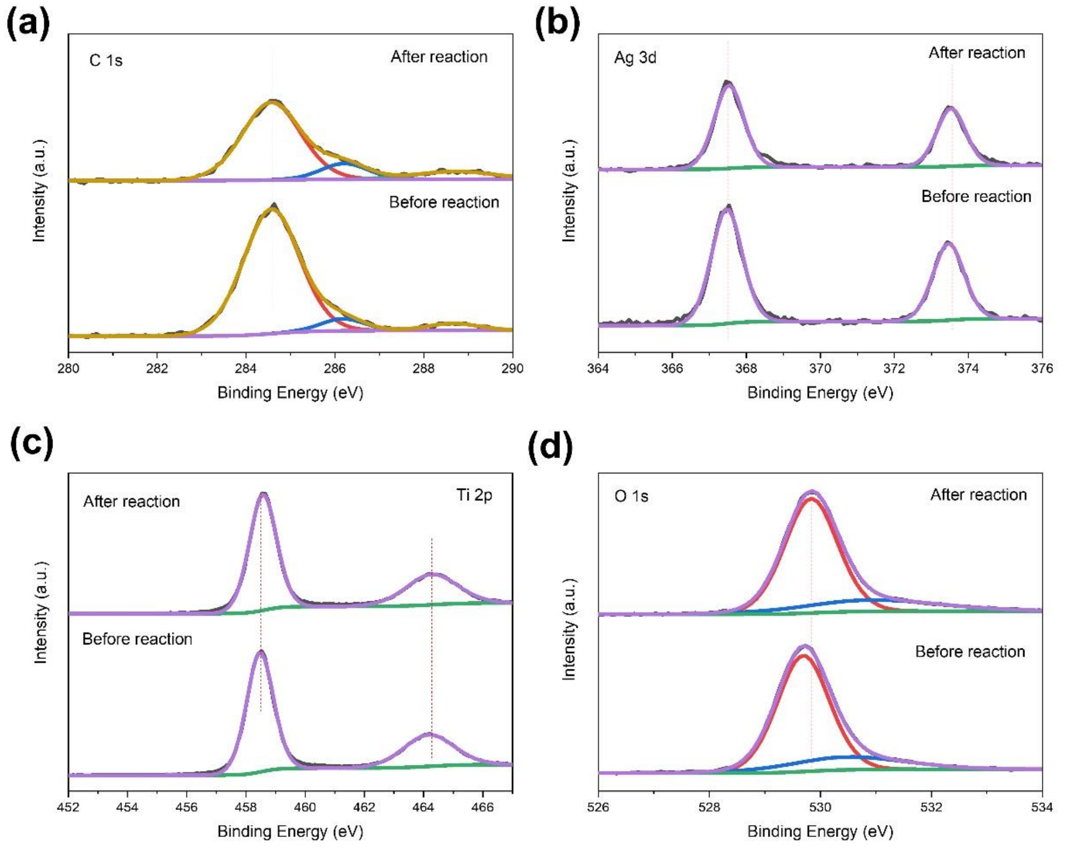

Figure S6 High-resolution XPS of C 1s (a), Ag 3d (b), Ti 2p (c) and O 1s of the Ag_{33} sintered TiO_2 samples before and after reaction.

Table S1 Quantification analysis derived from the survey XPS of Ag_{33}/TiO_2 samples before and after calcination.

Sample	Ag (At%)	Ti (At%)	Ag/Ti
Before calcination	1.09	98.91	0.011
After calcination	0.57	99.43	0.006
